# An essential role of disulfide bonds for the hierarchical self-assembly and underwater affinity of CP20-derived peptides

**DOI:** 10.3389/fbioe.2022.998194

**Published:** 2022-10-12

**Authors:** Baoshan Li, Junyi Song, Ting Mao, Ling Zeng, Zonghuang Ye, Biru Hu

**Affiliations:** ^1^ College of Science, National University of Defense Technology, Changsha, China; ^2^ Logistics Center, National University of Defense Technology, Changsha, China

**Keywords:** barnacle cement protein, BalCP20-derived peptides, self-assembly, wet adhesion, disulfide bonds

## Abstract

Barnacles are typical fouling organisms strongly adhere to immersed solid substrates by secreting proteinaceous adhesives called cement proteins (CPs). The self-assembly of the CPs forms a permanently bonded layer that binds barnacles to foreign surfaces. However, it is difficult to determine their natural structure and describe their self-assembly properties due to the abundance of cysteines in whole-length CP20. A putative functional motif of *Balanus albicostatus* CP20 (BalCP20) was identified to present distinctive self-assembly and wet-binding characteristics. Atomic-force microscopy (AFM) and transmission electron microscope (TEM) investigations showed that wildtype BalCP20-P3 formed grain-like spindles, which assembled into fractal-like structures like ears of wheat. SDS-PAGE, AFM, and LSCM showed that DTT treatment opened up disulfide bonds between cysteines and disrupted fractal-like structures. Additionally, these morphologies were abolished when one of the BalCP20-P3 four cysteines was mutated by alanine. Circular dichroism (CD) results suggested that the morphological diversity among BalCP20-P3 and its mutations was related to the proportion of *α*-helices. Finally, quartz crystal microbalance with dissipation (QCM-D) detected that BalCP20-P3 and its mutations with diverse self-assemblies occupied different affinities. The above results demonstrated that cysteines and disulfide bonds played a crucial role in the self-assembly and wet binding of BalCP20-P3. The work provides new ideas for the underwater bonding of BalCP20 and developing new bionic underwater adhesives.

## 1 Introduction

Biofouling occurs on the surface of all marine facilities by microorganisms, animals, and plants, which increases ship resistance, fuel consumption, and severe metal corrosion and causes great economic costs (Townsin, 2003; Schultz, 2007; Blackwood et al., 2017). Barnacles are renowned for their ability to maintain strong adhesion throughout their adulthood among those fouling organisms (Cleverley et al., 2021). Barnacles strongly adhere to immersed solid substrates using a mixture of CPs and self-assembles into a permanently bonded layer (Kumar et al., 2020). Their substrates are composed of CPs that bind to foreign surfaces even after they are dead and lost all their soft bodies. Hence, exploring the molecular mechanism of barnacle CPs is vital for developing new technology for either antifouling or underwater adhesive.

So far, six CPs have been identified by their apparent molecular weights: CP100, CP52, CP43 (used to be CP68), CP20, CP19, and CP16 (Mori et al., 2007; Urushida et al., 2007; Kamino et al., 2012; Kamino, 2016; So et al., 2016; Lin et al., 2021). These CPs have different spatial distribution and diverse functions. For example, insoluble proteins CP100 and CP52 may provide bulk properties in the barnacle cement. CP19, CP20, and CP43 have been speculated to be interfacial proteins and associate with surface functions, whereas CP16 is a minor constituent and shares homology with lysozyme-like enzymes. It is proposed to remove biofilms from the substratum and/or protect cements from microbial degradation (Tilbury et al., 2019). Moreover, CP20 is found to have adsorption activity to calcite, indicating its dual function for surface binding and biomineralization (Mori et al., 2007).

Kamino suggested that highly abundant Cys residues in *Megabalanus rosa* CP20 form intramolecular disulfide bonds, which are essential for the proper folding of the monomeric protein structure (Mori et al., 2007). NMR and molecular dynamics (MD) simulation results show that rMrCP20 contains three main folded domain regions intervened by two dynamic loops, resulting in multiple protein conformations in equilibrium. Besides, 12 out of 32 Cys in the rMrCP20 sequence engage in forming disulfide bonds (Mohanram et al., 2019). Kumar also used MD simulations to investigate the molecular interactions between rMrCP20 and calcium carbonate. Ca^2+^ and CO_3_^2-^ are sequestered by protein-charged surfaces (So et al., 2015). Breakthroughs have been made by the Ali Miserez group more recently. They observed the CaCO_3_ mineralization pathway regulated by rMrCP20 as well as the adhesive nanofibrils formed by its self-assembly. However, most studies focus on recombinant *Megabalanus rosa* CP20. CP20 from other species is rarely studied.

Our previous study expressed recombinant *Balanus albicostatus* CP20 in *E.Coli*. Purified proteins separate from the solution and form significant flocculent precipitation after being stored at 4°C (data not shown) although with a high expression yield. Since there are 18 cysteines in the BalCP20 sequence, intramolecular or intermolecular disulfide bonds are likely to form, as reported by Kamino et al. (Mori et al., 2007; Mohanram et al., 2019). These, disulfide bonds are essential for the proper folding of monomeric proteins. In the meanwhile, misplacing disulfide bonds causes protein misfolding and precipitation. Since the native state of disulfide bonds in CP20 was not clear, we suspended our study on whole-length BalCP20 and turned to explore the functions and properties of truncated peptides. We employed similar strategy as Kamino et al. did with MrCP-20 (kamino, 2001), and divided BalCP20 into four repeats according to the conserved cysteines. These derived peptides are constituent elements of whole-length BalCP20, as well as potential functional units. Take another typical underwater adhesive called mussel foot protein 2 (MFP2) as a reference, the cysteines in its EGF calcium-binding domain (EGF-CBD) helped it maintain the correct tertiary structure and allow it to effective bind calcium ion by forming intramolecular disulfide bonds (Hwang and Waite, 2012). During the process of our research, we found that the third part of BalCP20 (BalCP-P3) has a unique self-assembly behavior and forms distinct morphology. Hence, BalCP20-P3 peptides were likely to be the key to deciphering CP20 functions and mechanisms, especially its biomineralization properties.

AFM and TEM investigations showed that BalCP20-P3 formed grain-like spindles hierarchically assembled into fractal-like structures like the ears of wheat. SDS-PAGE, AFM, and LSCM showed that DTT treatment opened disulfide bonds between cysteines and disrupted fractal-like structures (ears of wheat). Four mutants of BalCP20-P3 (C→A) were designed to validify the essential role of cysteines and disulfide bonds in the CP20 self-assembly. Grain-like spindles and ears-of-wheat-like structures were abolished when one of the four cysteines in BalCP20-P3 was mutated by alanine. Circular dichroism (CD) results indicated that the secondary structure proportion of BalCP20 was substantially different. The unique morphology of BalCP20-P3 was manifested in the abundance of *α*-helices.

Finally, the detection of quartz-crystal microbalance with dissipation (QCM-D) showed that BalCP20-P3 and its mutations with diverse self-assemblies had different wet bindings. Cysteines and disulfide bonds played a crucial role in the self-assembly and wet binding of BalCP20-P3. The work provides new ideas for the underwater bonding of BalCP20 and developing new bionic underwater adhesives.

## 2 Materials and methods

### 2.1 Materials

Chemically synthesized peptides (purity is >95%); Milli-Q water (Milli-Q system, Millipore, Bedford, MA); Dithiothreitol (DTT); Thioflavin T (ThT) (Sangon Bioengineering (Shanghai) Co., Ltd.; Tricine SDS Sample Buffer; SeeBlueTM Plus2 Prestained Standard; NovexTM 10–20% Tricine Gel; Tricine SDS Running Buffer (10×) (ThermoFisher SCIENTIFIC, United States).

### 2.2 Preparation of peptides

**FIGURE 1:**
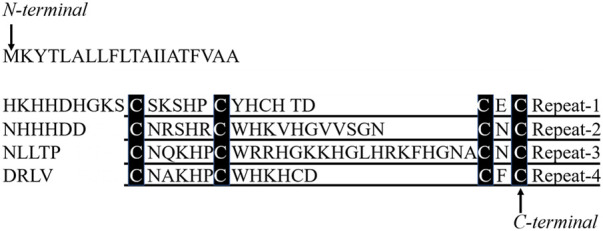
Four repetitive units in 20 kDa cement protein and peptides derived from the primary structure and sequence comparison of BalCP20-P3 and EGF-CBD. Four repetitive sequences in 20 kDa cement protein, repeat-1 to repeat-4, are aligned. Amino acid residues conserved in all repeats are denoted with black boxes.

Peptides designed in the work were derived from the third repeat unit in the primary structure of BalCP20 (see Ref. (He et al., 2013). for the amino acid sequence). The full-length amino acid sequence of BalCP20 was obtained from the NCBI (National Center for Biotechnology Information) database and divided into four repeats according to the conserved cysteines (see Figure 1). Based on our previous studies, especially microscopic observations, the P3 sequence was likely to be the functional unit of BalCP20. Series mutants of BalCP20-P3 were designed by alanine scanning to investigate the effects of the number and position of cysteines. Designed peptides were synthesized by Fmoc solid-phase (Kim et al., 2021) and purified by reverse-phase high-performance liquid chromatography using a Zorbax 300SB-C18 column (4.6 * 150 mm; Agilent Technologies, Palo Alto, CA). Elution was conducted with a water-acetonitrile linear gradient [100% (v/v) acetonitrile] containing 0.1% (v/v) trifluoroacetic acids. The molecular mass of the purified peptide was confirmed by liquid chromatography/mass spectroscopy using a Water 2695 HPLC Separations Module (Waters Alliance, United States) (S1-S5). The synthesized peptides were dissolved in ultrapure water (Milli-Q system, Millipore, Bedford, MA) and stored at 4°C.

### 2.3 Visualization with SEM, TEM, AFM, and LSCM

Peptide powders were dissolved in Milli-Q at 4°C before SEM analysis. Place 10-μL BalCP20-P3 (1 mg/ml) peptide solutions on a clean silicon wafer and dry at ambient temperatures. Samples on silicon wafers were then observed by an S-4800 scanning electron microscope (Hitachi, Japan).

10-μL peptide Milli-Q solutions were placed on the newly peeled surface of the mica wafer and dried at ambient temperatures for AFM analysis. AFM images were captured in the tapping mode by a NanoScope-ScanAsyst in the air mode. The wks controller (Bruker Corporation, DE) and intelligent scanning (ScanAsyst in AIR) used a silicon probe with a cantilever length of 115 μm and a spring constant of 0.4 N/m. Images with a scanning range of 0.6–8 μm were taken at a scanning rate of 1 Hz with 256 lines per image. LSCM was photographed with TCS-SP8 (Leica MICROSYSTEMS, DE), and the photographed results were processed with ImageJ software.

10-μL peptide Milli-Q solutions were placed on a 200-mesh copper-mesh support film (Beijing Zhongjingkeyi Technology Co., Ltd.) for the transmission electron microscopy (TEM) analysis. Copper meshes were kept wet for 5 min. Then 10-μL aliquot of uranyl acetate (mass fraction 2%, Hubei Chushengwei Chemical Co., Ltd.) was stained for 5 min and dried at room temperatures. TEM images were recorded using a transmission electron microscope TEM (HT-7700, Hitachi, Japan) (Sonzini et al., 2015).

### 2.4 DTT treatment and SDS-PAGE

SDS-PAGE was performed according to the method of Gao et al. (Gao et al., 2010) with modification in electrophoresis apparatuses and conditions: 10-μL BalCP20-P3 samples prepared as described above were mixed with an equal volume of gradient DTT (4–20 mm, Sangon Bioengineering (Shanghai) Co., Ltd.) and 20-μL Tricine SDS sample buffer (2×) (Thermo SCIENTIFIC, United States). Then place in a water bath at 70°C for 10 min. After that, samples were electrophoresed with NovexTM 10–20% Tricine Gel (Thermo SCIENTIFIC, United States) at 200 V, 120 mA for 10 min and 200 V, 80 mA for 30 min. Stain with Coomassie brilliant blue G-250 for 5 h, and decolorize with 10% acetic acid solutions.

### 2.5 Circular dichroism (CD) and Thioflavin T (ThT)

CD data were recorded on a BRIGHTTIME Chirascan, JASCO810, Jasco-815 spectrometer, measuring from 260 to 190 nm at 25°C (Sonzini et al., 2015). Spectra Manager software was used to smooth CD data, which were saved and uploaded to http://dichroweb.cryst.bbk.ac.uk. Wavenumber ranges of 190–260 nm were selected to calculate the relative content of the secondary structure (Nakano and Kamino, 2015). Three independent samples were analyzed, and data were averaged.

A Thioflavin T (ThT, Sangon Bioengineering (Shanghai) Co., Ltd.) test was conducted to determine whether CP20-P3 underwent Amyloid-like self-assembly (Matiiv et al., 2020). A 20-mm ThT aqueous concentrate was diluted 1,000-fold with 50-mm potassium phosphate buffer (at pH 6.0) immediately before use. Aliquots of the peptide concentrate (0.2–4 mg/ml) were mixed with an equal volume of diluted ThT solutions, and the mixture was then incubated at 4°C for 30 min. Fluorescence was measured with a FLUOROSKAN ASCENT FL spectrometer (Thermo SCIENTIFIC, United States) at 25°C with an excitation wavelength of 430 nm and an emission wavelength of 481 nm. The fluorescence intensity of the blank solution without peptides was subtracted as a background. Peptides with fluorescence intensities of >0.5 were defined as ThT binding in the work (Nakano and Kamino, 2015).

### 2.6 Dissipative quartz crystal microbalance (QCM-D)

Gold-coated quartz crystal sensors with fundamental frequency (5 MHz) QSX 301 (Biolin Scientific, Sweden) were used as the wet binding platforms for BalCP20-P3 and its mutants. Gold sensors were first cleaned with deionized water of 5:1:1 (v/v), 25% (v/v) ammonia water, and 30% (v/v) hydrogen peroxide for 10 min in the water bath at 75°C. Then they were placed into different QCM-D chambers (Kohn et al., 2020). Changes in frequencies (△*f*, Hz) and dissipation (△*D*, 1 × 10^−6^) at the third, fifth, seventh, ninth, and 11th overtones were monitored at 25°C in all chambers using a Q-Sense E4 QCM-D system (Biolin Scientific, Stockholm, Sweden). All flow rates for solutions were at 0.1 ml/min, and all volumes given are on a per-chamber basis (Lozeau et al., 2015). A Milli-Q flush was used to obtain a final baseline after the frequency and dissipation were stabilized, and QSoft 401 was used for data recording and proceeding.

## 3 Results

### 3.1 Morphological observation by SEM/TEM/AFM

**FIGURE 2:**
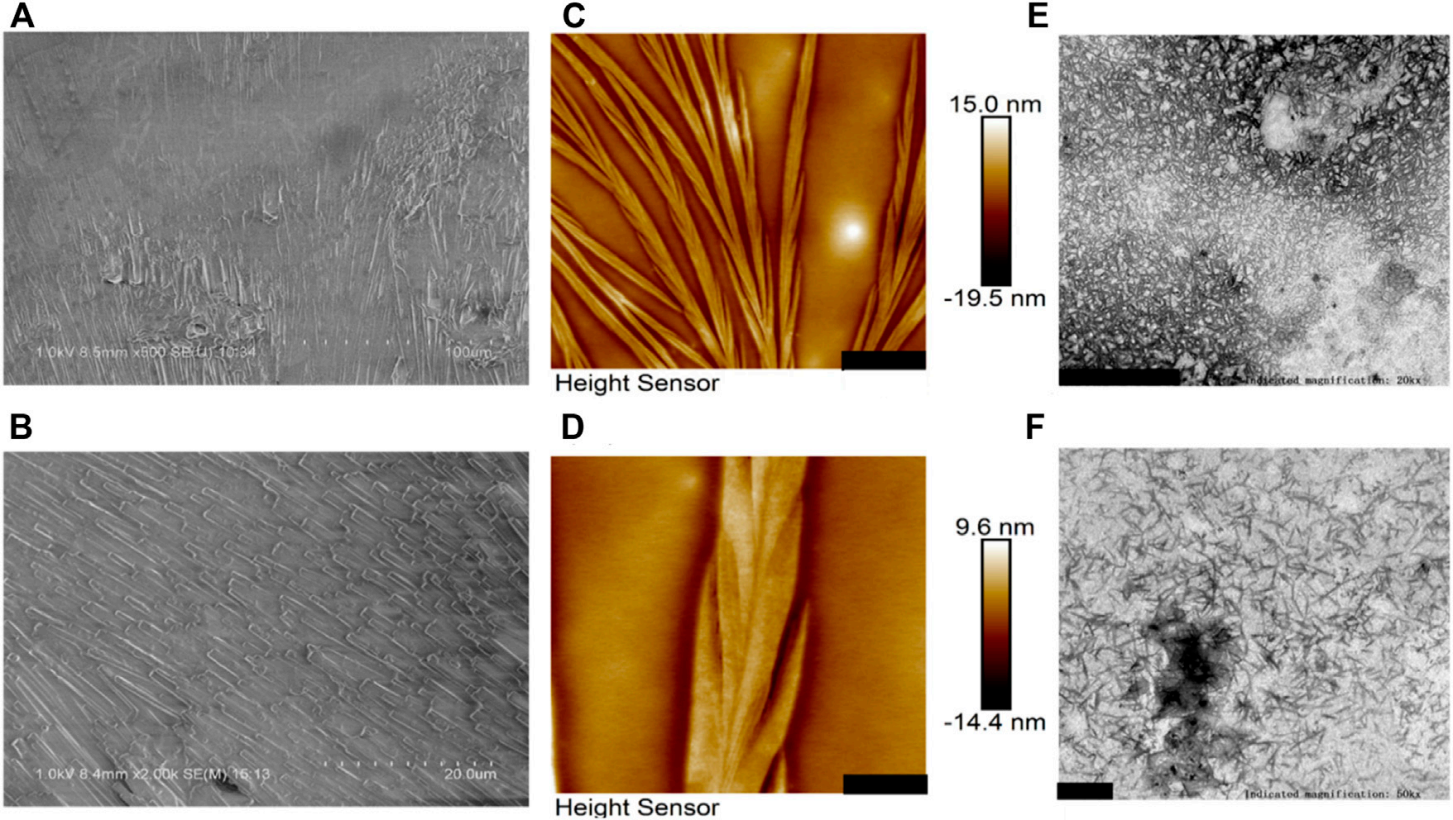
Microscopic characterizations of BalCP20-P3. **(A,B)** represent scale bars = 100 and 20 μm for SEM of BalCP20-P3, respectively; **(C,D)** represent scale bars = 3 and 1 μm for AFM of BalCP20-P3, respectively; **(E,F)** respresent scale bars = 1 and 200 μm for TEM of BalCP20-P3, respectively.

Figure 2 presents morphological observations of BalCP20-P3 dissolved in Milli-Q. SEM analysis reveals the formation of compact stacked stick within hours after the BalCP20-P3 were dissolved in Milli-Q. The stick are about one micron in diameter, and almost no amorphous structures were observed within the entire sample (see Figures 2A,B). Samples are analyzed by high-resolution AFM and TEM to discern the structural characteristics of stacked peptides. Assembled peptide structures deposited on mica form regular wheat spike-like structures with a 3D conformation. AFM analysis indicates that wheat spike-like structures are about 1 μm in diameter, while the length and width of their subunits like wheat grains vary from hundreds of nanometers to micrometers (see Figures 2C,D). Moreover, TEM analysis showed that the peptides of BalCP20-P3 assemble into short spindles about 100–200 nm, which are cross-linked end-to-end (see Figures 2E,F). It is speculated that the differences in sample preparations result in the diversity of peptide morphologies.

Samples are dropped on copper meshes for TEM. Peptides sinking into micropores are not influenced by the changes in aqueous tensions during desiccation at room temperatures. However, liquid flow acts on the self-assembly of peptides during desiccation for SEM, which gathers samples in certain regions and forms larger grains and wheat spikes. As to the difference between SEM and AFM results, the vacuum operation conditions of SEM contribute to the transition from wheat spikes into stacked sticks.

### 3.2 SDS-PAGE/AFM/LSCM of BalCP20-P3 after DTT treatment

**FIGURE 3:**
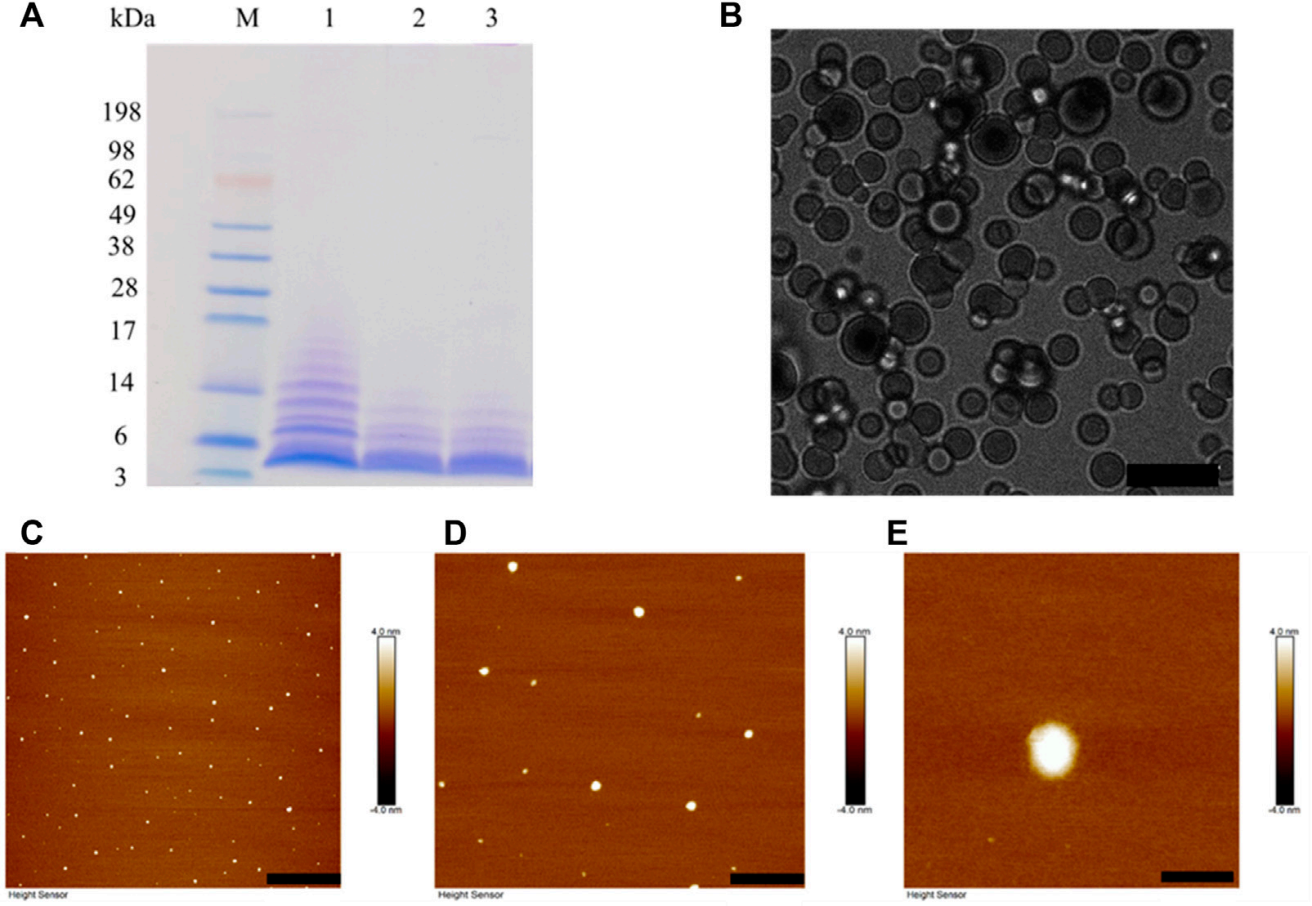
BalCP20-P3 after DTT treatment. **(A)** SDS-PAGE of BalCP20-P3±DTT, where M is Mark protein; Lane 1 is BalCP20-P3 of -DTT; Lane 2 is BalCP20-P3 treated by 1 mM DTT; Lane 3 is BalCP20-P3 treated by 5 mM DTT (BalCP20-P3 _MW_ = 3.35389 kDa). **(B)** LSCM of BalCP20-P3 after DTT treatment. BalCP20-P3 exhibits a regular spherical shape, and scale bar = 10 μm. **(C**–**E)** AFM of BalCP20-P3 after DTT treatment, where spherical particles are evenly distributed in the field of view, with scale bar = 3 μm, 1 μm, and 200 nm, respectively.

BalCP20-P3 solutions are treated with different DTT concentration and evaluated by SDS-PAGE (see Figure 3A). Obvious smears exist in the lanes for BalCP20-P3 treated with low/no DTT concentration. As DTT concentration goes up, smears gradually disappear and the bands enrich around 3 kDa. Disulfide bonds among BalCP20-P3 are disrupted, with oligomers/monomers released. Correspondingly, the grain-like or wheat spike-like morphology of BalCP20-P3 also disappears. Both LSCM and AFM detect spherical structures in DTT-treated samples. Since these spherical particles are flowing in droplets, it is difficult for LSCM to determine a focal plane (see Figure 3B). AFM describes that spherical particles with diameters of approximately 150 nm (see Figures 3C–E) are evenly distributed in the entire area.

#### 3.2.1 AFM/CD/ThT of BalCP20-P3 mutants

**FIGURE 4:**
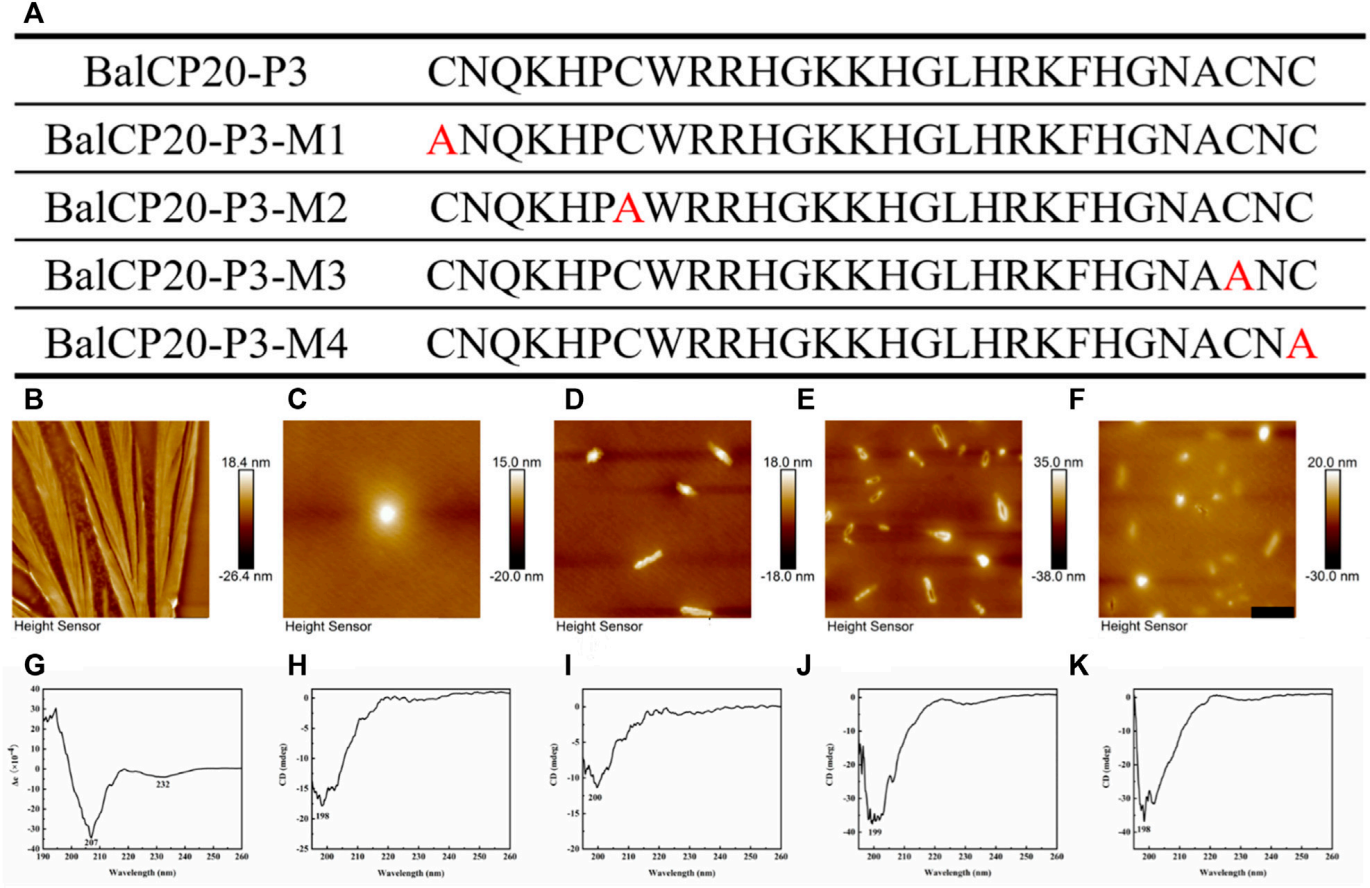
Sequence design, AFM, CD of BalCP20-P3 and its mutants. **(A)** BalCP20-P3 and its mutant amino acid sequence design. **(B)** Mutants mutated amino acid residues are marked in red, and BalCP20-P3 presents a wheat spike-like structure. **(C)** Spherical self-assembly of BalCP20-P3-M1. **(D)** BalCP20-P3-M2 and BalCP20-P3-M3 **(E)** are rod-like structures of varying lengths. **(F)** BalCP20-P3-M4 has either spherical or rod-like structures. The scale bars of **(B**–**F)** are 1 μm. **(G)** CD of BalCP20-P3. **(H)** CD of BalCP20-P3-M1. **(I)** CD of BalCP20-P3-M2. **(J)** CD of BalCP20-P3-M3. **(K)** CD of BalCP20-P3-M4. The abscissa is the wavelength (190–260 nm) and the ordinate is the absorbance.

Four mutants are designed by changing one of the four cysteines in BalCP-P3 to alanine to verify the fundamental role of cysteines or disulfide bonds (see Figure 4A). Firstly, different self-assembly properties between BalCP20-P3 and mutants are observed by AFM. Wildtype BalCP20-P3 has a delicate wheat-spike hierarchical structure. However, these morphological features are entirely abolished when one cysteine is mutated. AFM observed spheres with diameters of approximately 500 nm in BalCP20-P3-M1 and BalCP20-P3-M4 samples (see Figures 4C,F) as well as short rods with a length of about 1 micron in BalCP20-P3-M2-4 (see Figures 4D–F). Furthermore, circular-dichroism spectra are conducted to explain their different self-assembly behaviors in the secondary structure.

Unordered and turn structures show negative peaks near 195 nm, while *β*-sheets generally show positive and negative peaks near 195 and 220 nm, respectively. *α*-helix structures show positive and negative peaks near 190 and 222 nm, respectively (Barlow et al., 2010). Table 1 presents the proportions of secondary structures of BalCP20-P3 and its mutants. The secondary structure of BalCP20-P3 is dominated by *α*-helix, and those of the mutants of BalCP20-P3 are dominated by *β*-sheets with disordered distribution. Normalized root mean square deviations (NRMSDs) of BalCP20-P3 and its mutants below 0.15 indicate acceptability (Baker and Garrell, 2004).

**TABLE 1:** Secondary structure ratios of BalCP20-P3 and its mutants.

Sample	α-helix	3_10_-α-helix	β-sheet	β-turn	PP2	Unordered	NRMSD
BalCP20-P3	0.348	0.170	0.020	0.122	0.196	0.129	0.022
BalCP20-P3-M1	0.004	0.033	0.144	0.153	0.165	0.501	0.072
BalCP20-P3-M2	0.003	0.040	0.216	0.144	0.139	0.458	0.099
BalCP20-P3-M3	0.002	0.032	0.279	0.115	0.130	0.442	0.145
BalCP20-P3-M4	0.005	0.111	0.045	0.181	0.151	0.507	0.139

NRMSD, are shown in italics, and that below 0.15 indicates a credible result.

### 3.3 Characterizations of underwater wet bindings of BalCP20-P3 and its mutants

**FIGURE 5:**
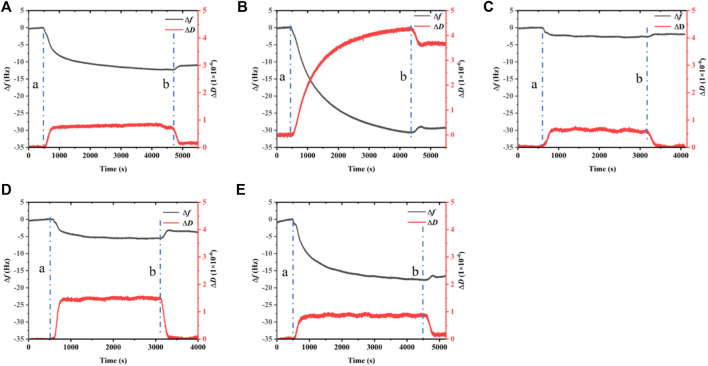
Dissipation, frequency of BalCP20-P3 and its mutants on gold-coated quartz crystal sensors. **(A)** BalCP20-P3. **(B)** BalCP20-P3-M1. **(C)** BalCP20-P3-M2. **(D)** BalCP20-P3-M3. **(E)** BalCP20-P3-M4. Changes in frequencies (Hz) in black are shown on the primary axis, and changes in dissipation (1 × 10^−6^) in red are shown on the secondary axis. **(A)** Flow of BalCP20-P3 and its mutants solution; **(B)** Million-Q rinse.

**FIGURE 6:**
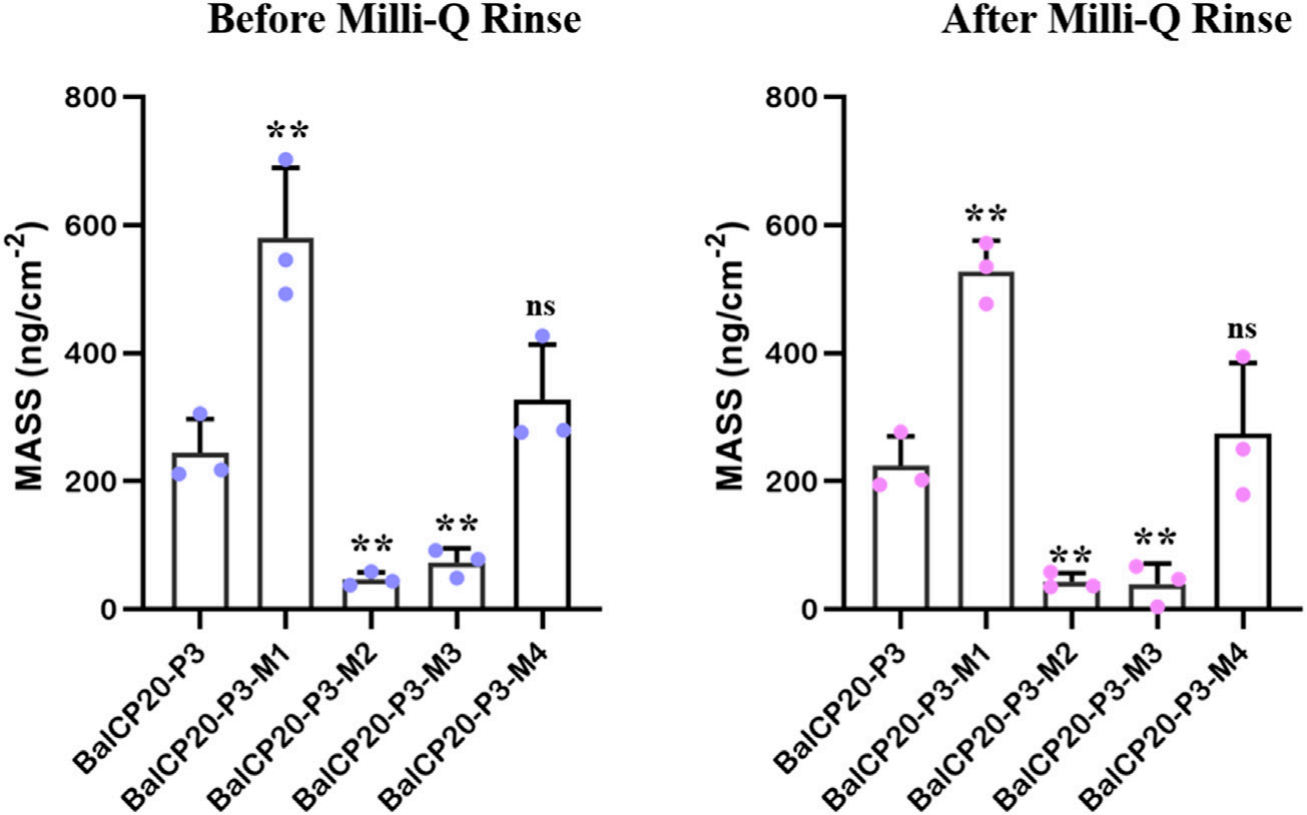
Unit area adsorption amount of BalCP20-P3 and its mutants on gold-coated quartz crystal sensors. Data are presented as mean values ±SD for *n* = 3 independent experiments. *p*-values (from a two-sided unpaired *t*-test): * *p* < 0.05; ** *p* < 0.01; *** *p* < 0.001; all groups are compared to BalCP20-P3.

QCM-D is used to record the frequency and dissipation changes in BalCP20-P3 and its mutants on the gold surface (see Figure 5). △m is 39.56 ± 32.16 and 527.69 ± 47.87 ng per crystal due to polypeptides according to the Sauerbrey calculation ( $△m=-\frac{\left(C.△f\right)}{n}$ *c* = 17.7 ng Hz ^−1^ cm ^−2^; *n* = 1) (see Figure 6 and Table 2). Attached masses decrease with the increased frequency. When peptide solutions are introduced, △*f* rapidly decreases corresponding to △*m* from 46.89 ± 11.02 to 580.03 ± 108.97 ng (see Figure 6; Table 2) bound to the surface. The variation of △*D* is large ((-32 to 0) × 10^−6^) during the process.

**TABLE 2:** Mass of bindings of BalCP20-P3 and its mutants.

Sample	Before Milli-Q rinse (ng/cm^2^)	After Milli-Q rinse (ng/cm^2^)
BalCP20-P3	244.69 ± 52.40	224.54 ± 45.71
BalCP20-P3-M1	580.03 ± 108.97	527.69 ± 47.87
BalCP20-P3-M2	46.89 ± 11.02	43.39 ± 12.95
BalCP20-P3-M3	73.11 ± 21.93	39.56 ± 32.16
BalCP20-P3-M4	327.55 ± 85.75	274.73 ± 109.40

Data are presented as mean values ±SD, for *n* = 3 independent experiments.

When Milli-Q is introduced to the bounded gold surface of BalCP20-P3, △*m* decreases from 244.69 ± 52.40 to 224.54 ± 45.71 ng (see Figure 6; Table 2). The variation of △*D* is large (approximate (−12 to 0) ×10^−6^) during this process. Similarly, when Milli-Q is introduced to the bound surface of BalCP20-P3 mutants, △m decreases from 580.03 ± 108.97 to 527.69 ± 47.87 ng for BalCP20-P3-M1, 46.89 ± 11.02 to 43.39 ± 12.95 ng for BalCP20-P3-M2, 73.11 ± 21.93 to 39.56 ± 32.16 ng for BalCP20-P3-M3, and 327.55 ± 85.75 to 274.73 ± 109.40 ng for BalCP20-P3-M4. The variation of △*D* is large [approximate (−30 to 0) × 10^−6^] during this process (see Figure 1).

BalCP20-P3 and mutants are bound to gold surfaces in QCM-D results. Relatively, the attachment of BalCP20-P3-M1 and BalCP20-P3-M4 increases △*m* and △*D* and decreases △*f*, indicating that more peptides are bound to gold surfaces. BalCP20-P3-M2 and BalCP20-P3-M3 adhere to the least amount, and BalCP20-P3 is centered.

## 4 Discussion

A novel peptide derived from the BalCP20 amino-acid sequence was studied in the work. CP20 played a role in binding and biomineralization, and we speculated that CP20 directly participated in forming the calcareous chassis through its specific conformation binding to the ubiquitous calcium ions in seawater. As a natural comparison, conserved cysteines in the calcium-binding domain (EGF) help mussel foot protein MFP-2 maintain the correct 3D structure by forming intramolecular disulfide bonds to bind calcium ions. During the study, we found that BalCP20-P3 has a unique self-assembly behavior, and the number and position of cysteines have a significant effect on its self-assembly and wet-binding ability. Therefore, P3 was used to replace CP20 for research. Peptides presented distinct self-assembly. Firstly, peptides were assembled into spindles with lengths of about hundreds of nanometers. Subsequently, these spindles further aggregated into grain-like structures and even wheat-spike-like hierarchical morphology. Thirdly, this unique self-assembly of BalCP20-P3 was related to its underwater binding ability.

BalCP20 and BalCP20-P3 were speculated to undergo an amyloid-like fibril formation process, like CP19 (Liu et al., 2017) and CP52 (Nakano and Kamino, 2015). However, no significant fluorescence was detected by Thioflavin-T tests. Therefore, the CD was employed to decipher the self-assembly mechanism of BalCP20-P3. Self-assembly was a spontaneous organization with simple molecular buildings blocked into supramolecular structures.

Typical noncovalent interactions such as hydrogen bonding, electrostatic attraction, van der Waals forces, and aromatic contacts directed this process and endowed peptides with distinct secondary structures (Aida et al., 2012). *α*-helices and *β*-sheets were the two most abundant structural motifs regulating the interfacial interactions between neighboring proteins (Richardson, 1981). H-bonding propagated in the axial direction within the helical axis and every N-H amide proton formed H-bonding with the amide C=O group located at the fifth position in helical peptides. Besides, the second type of prevalent intermolecular H-bonding observed in proteins involves amino-acid residues located at every first and fourth position, known as the 3_10_-*α*-helix (Mondal and Gazit, 2016).

As circular dichroism spectra were detected, there were 34.8% *α*-helices and 17% 3_10_-*α*-helices in BalCP20-P3. However, the proportion of helices in BalCP20-P3 mutants was rare (3–10%). It explained why the morphologies of BalCP-P3 and its mutations were so different (see Table 1). Since there was high helix content, BalCP-P3 molecules were twisted and their supramolecular structures looked like spindles. However, mutants of BalCP20-P3 were assembled into spheres or short rods without twists.

Typical seven amino acid model “c-d-e-f-g-a-b” existed in BalCP-P3, which contributed to forming helices. a and d represented hydrophobic amino acids, which provided a hydrophobic surface in the spiro-selective structure and acted as oligo domains with combined multiple helical peptides. e and g represented charged amino acids, which provided electrostatic action and promote helix formations. b, c, and f had little effect on the helical structure (Potekhin et al., 2001). If there were sticky ends at both ends of the helical peptide, such as cysteine (Depuydt et al., 2011), they would be connected and extend in lateral and longitudinal directions to form micron-scale self-assemblies.

**FIGURE 7:**
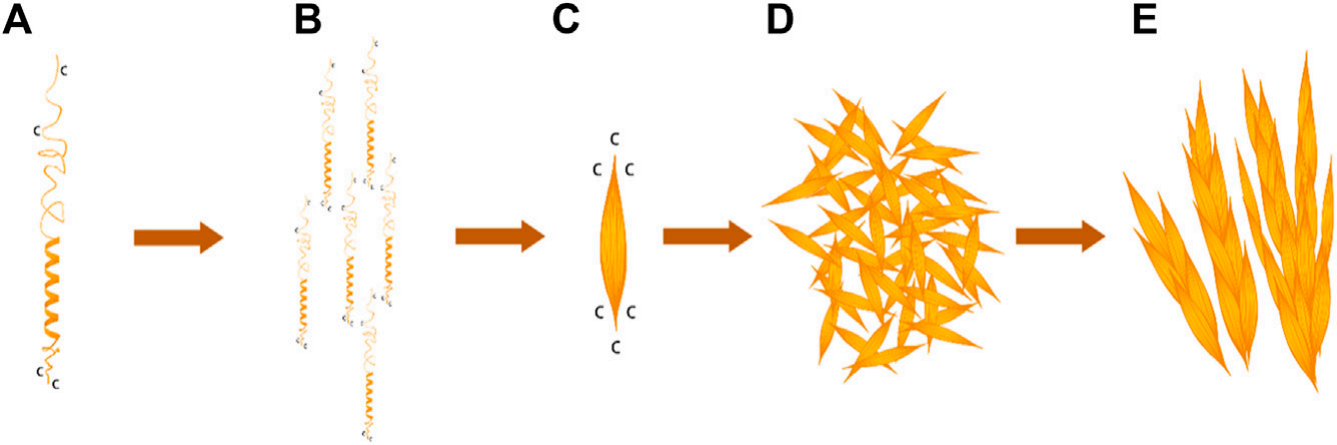
BalCP20-P3 self-assembly mechanism. **(A)** Spiral model of BalCP20-P3. **(B)** Monomer arrangement model of BalCP20-P3. **(C)** Spindle of BalCP20-P3. **(D)** Self-assembly model of BalCP20-P3 under TEM. **(E)** Model of the wheat spike structure of BalCP20-P3.

G-L-H-R-K-F-H in the BalCP20-P3 sequence was a typical heptad repeat in the work. H and K provided electrostatic force; F and L provided hydrophobic interfaces. BalCP20-P3 with seven amino acid model first formed helical units under electrostatic and hydrophobic interactions (see Figure 7A). These helical units cross-linked and extended transversely and longitudinally to form grain-like structures using disulfide bonds, as detected by TEM (see Figures 7E,F). These grain-like structures were gathered into micron-scale wheat-spike-like or stacked pipes in AFM and SEM assays.

BalCP20-P3 solutions were deposited on the mica for AFM sample preparation. As the water gradually evaporated at room temperatures, water flow was applied to the peptide assemblies. These spindles clumped to form larger structures-grain-like or wheat-spike-like structures (see Figures 7C,D) due to the differences in viscosities between the air and solutions (Chen et al., 2016). Since there was an additional vacuuming operation for SEM, the self-assemblies of BalCP20-P3 were more compact and closely gathered as stacked piles (see Figures 7A,B).

QCM-D results showed that the mutation of cysteine significantly affected the self-assembly morphologies of BalCP20-derived polypeptides and their underwater binding ability. The steric hindrance and electrostatic force of cysteines at both ends of the sequence were lower during the self-assembly process of BalCP20-P3, BalCP20-P3-M2, and BalCP20-P3-M3, which was more conducive to forming paired disulfide bonds. These paired disulfide bonds acted as sticky ends to constrain the steric distortion of the polypeptide molecule, which forms rod-like or spindle structures during self-assembly.

Cysteine at both ends was mutated to alanine in BalCP20-P3-M1 and BalCP20-P3-M4, resulting in the existence of disulfide bonds mostly in a single form. The connection mode of a single disulfide bond had a higher freedom degree of steric distortions, and more spherical structures formed during self-assembly. Therefore, BalCP20-P3-M1 and BalCP20-P3-M4 had more cysteine to form Au-S bonds when adsorbed on the gold-coated quartz crystal sensors. Besides, the binding was stronger and less likely to be washed away by water. Similarly, BalCP20-P3, BalCP20-P3-M2, and BalCP20-P3-M3 formed fewer Au-S bonds with relatively weak binding. BalCP20-P3 had 25% more cysteine, so stickiness was stronger than BalCP20-P3-M2 and BalCP20-P3-M3.

In summary, a potential function motif was found in the BalCP20 amino-acid sequence. Results bring new insights into the biochemical properties and wet binding mechanism of barnacle cement protein CP20 as well as the development of new-type bionic adhesives. The future work will study its self-assembly properties in conditions more similar to sea conditions (such as pH and ionic strength) to better understand the interaction mechanism of BalCP20 and guide its bionic applications.

## Data Availability

The raw data supporting the conclusions of this article will be made available by the authors, without undue reservation.
