# Supplementary material for: An essential role of disulfide bonds for the hierarchical self-assembly and underwater affinity of CP20-derived peptides
Source: Front Bioeng Biotechnol. 2022 Oct 12;10:998194. doi: 10.3389/fbioe.2022.998194 (PMC9597634; doi:10.3389/fbioe.2022.998194)
Supplement: Supplementary file 1 [file DataSheet1.PDF]

Supplementary Material

1.1 Supplementary Figures

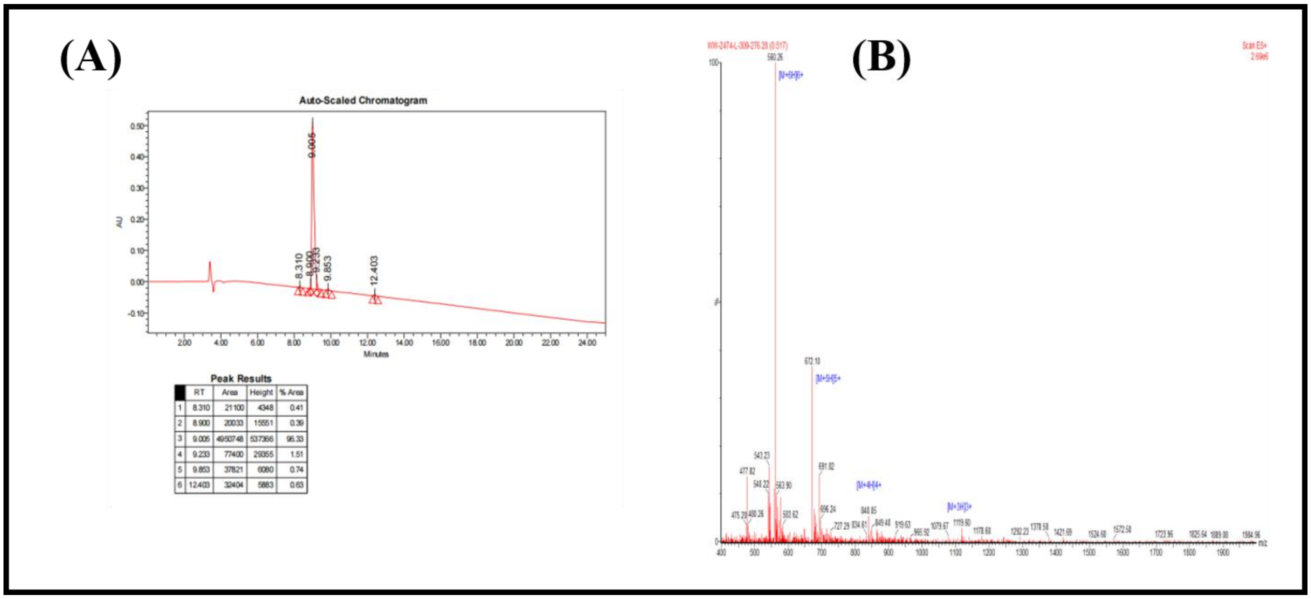

	RT	Area	Height	% Area
1	8.310	21100	4348	0.41
2	8.900	20033	15551	0.39
3	9.005	4950748	537366	96.33
4	9.233	77400	29355	1.51
5	9.853	37821	6080	0.74
6	12.403	32404	5883	0.63

Supplementary Figure 1. HPLC of BalCP20-P3 (A), MS of BalCP20-P3 (B)

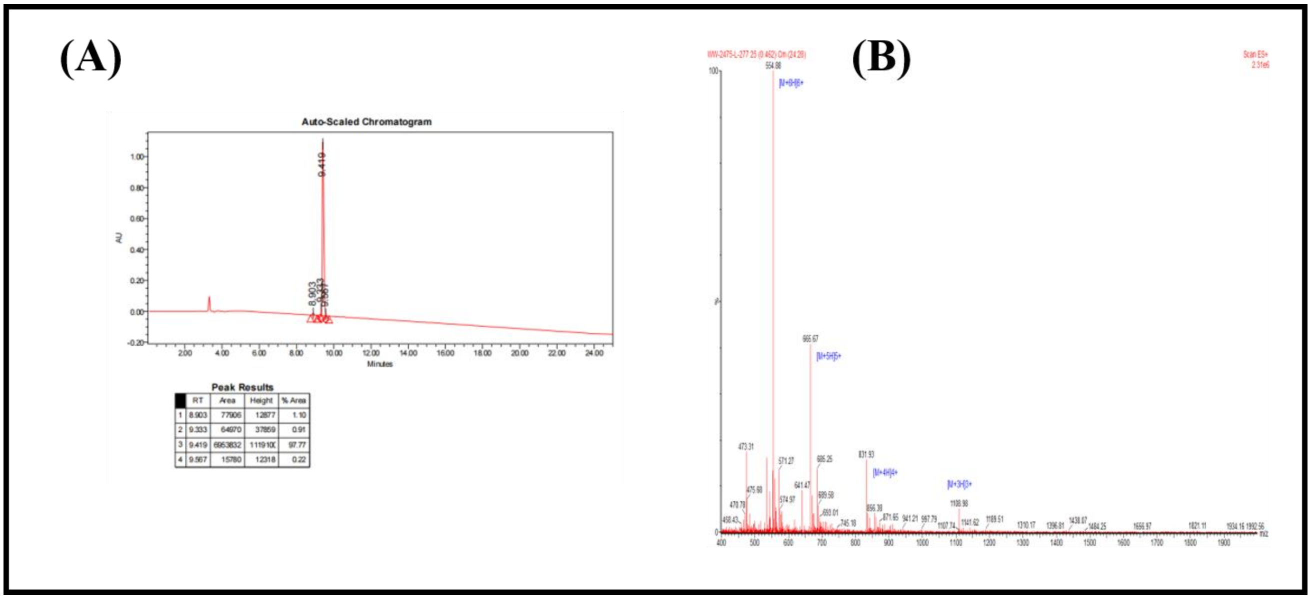

	RT	Area	Height	% Area
1	8.903	77906	12877	1.10
2	9.333	64970	37859	0.91
3	9.419	6953832	111910(	97.77
4	9.567	15780	12318	0.22

Supplementary Figure 2. HPLC of BalCP20-P3-M1 (A), MS of BalCP20-P3-M1 (B)

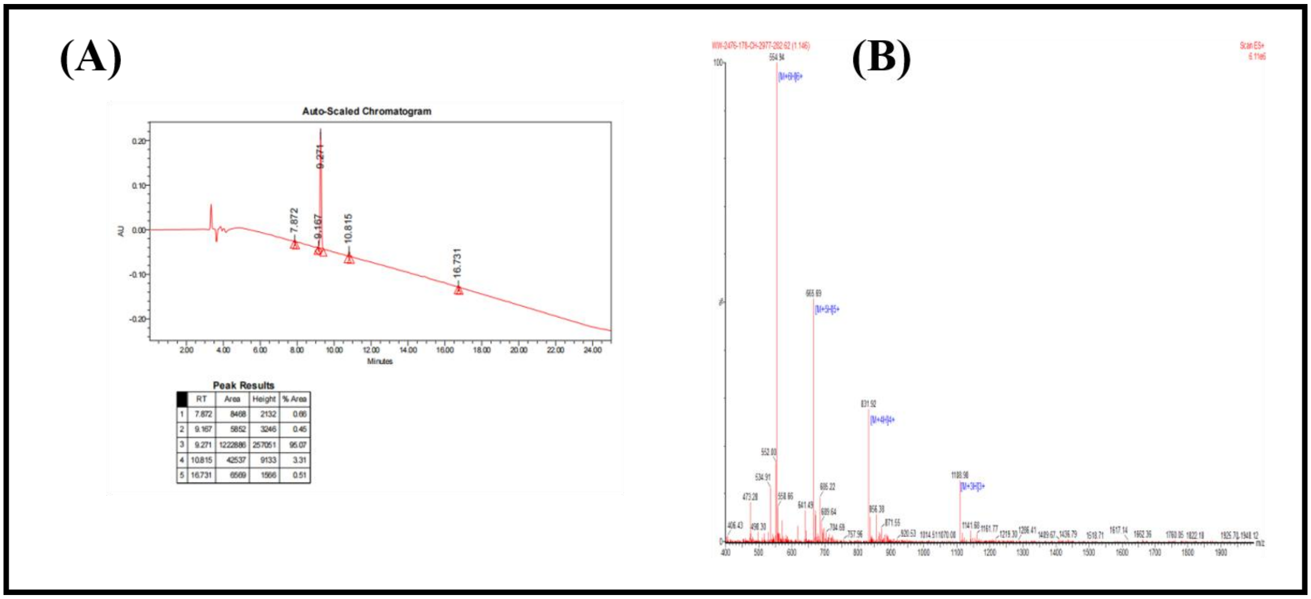

Supplementary Figure 3. HPLC of BalCP20-P3-M2 (A), MS of BalCP20-P3-M2 (B)

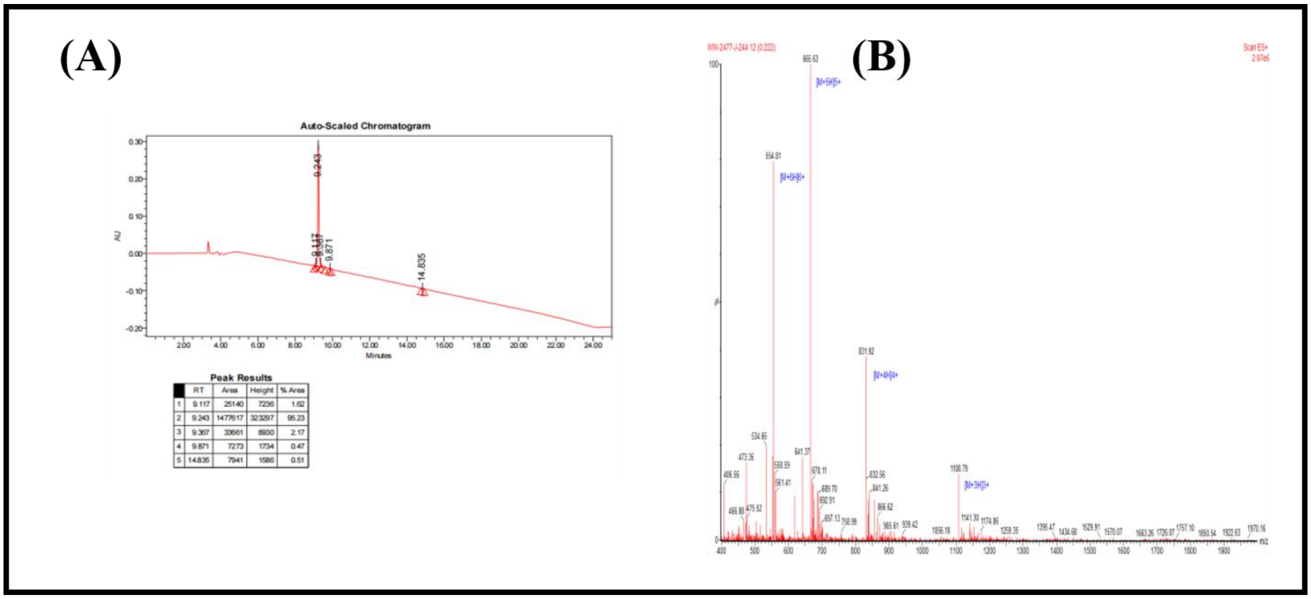

Supplementary Figure 4. HPLC of BalCP20-P3-M3 (A), MS of BalCP20-P3-M3 (B)

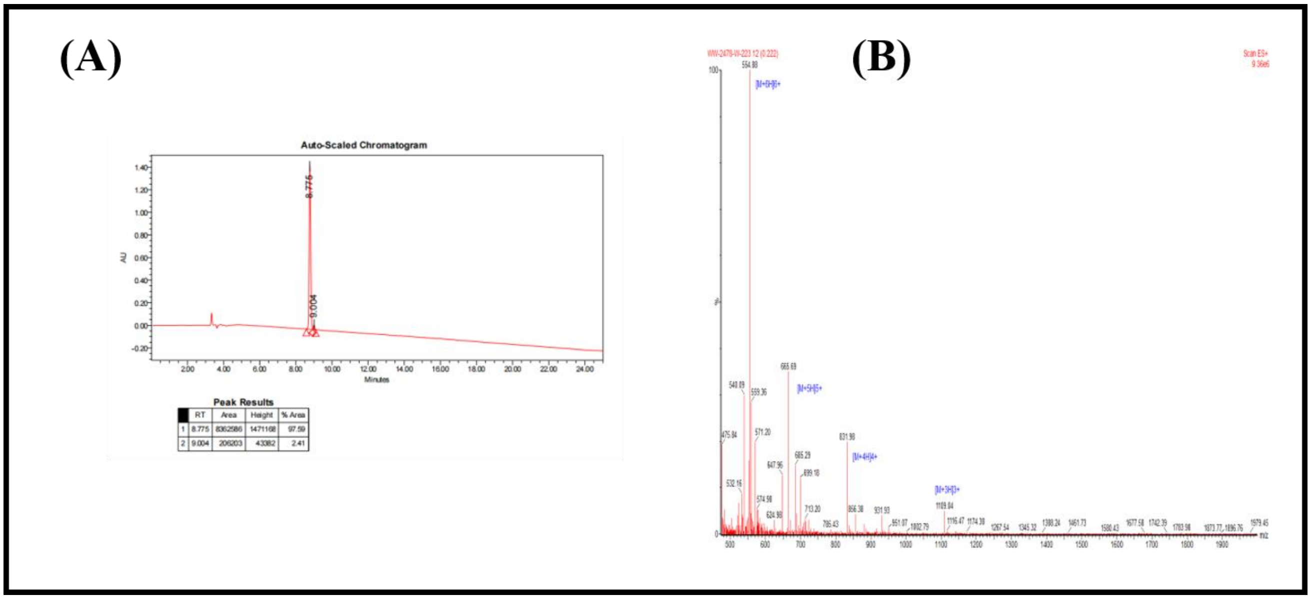

Supplementary Figure 5. HPLC of BalCP20-P3-M4 (left), MS of BalCP20-P3-M4 (right)
